# On the Involvement of BDNF Signaling in Memory Reconsolidation

**DOI:** 10.3389/fncel.2019.00383

**Published:** 2019-08-22

**Authors:** Maria Carolina Gonzalez, Andressa Radiske, Martín Cammarota

**Affiliations:** Memory Research Laboratory, Brain Institute, Federal University of Rio Grande do Norte, Natal, Brazil

**Keywords:** neurotrophin, memory reconsolidation, TrkB, BDNF, retrieval

## Abstract

When retrieval occurs concomitantly with novelty detection, mismatch perception or reactivation of conflicting information, consolidated memories can enter into a labile state, and to persist, must be restabilized through a protein synthesis-dependent reconsolidation process during which their strength and content can be modified. Extensive literature implicates brain-derived neurotrophic factor (BDNF), a key regulator of synaptogenesis and synaptic plasticity, in the acquisition, consolidation and extinction of several memory types. However, the participation of BDNF in memory reconsolidation has been less studied. In this review, we discuss recent reports supporting the involvement of BDNF signaling in reactivation-induced memory updating.

## Introduction

Memory consolidation is the time and protein synthesis-dependent stabilization process that takes place after learning to convert short-term memory into long-term memory. Consolidated memories are stable but mutable and can return to a labile state when activated during retrieval, requiring a restabilization phase known as reconsolidation to endure. Consolidation and reconsolidation share several molecular properties and result in persistent synaptic changes for memory storage. However, they are distinguishable processes that serve different biological purposes.

Brain-derived neurotrophic factor (BDNF) regulates neurogenesis, neuronal differentiation, maturation and survival during development (Park and Poo, 2013). BDNF is enriched in the adult’s brain hippocampus and cerebral cortex (Conner et al., 1997), where it exerts neuroprotective effect, enhances synaptogenesis and neurotransmission, and mediates activity-dependent synaptic plasticity (Poo, 2001; Panja and Bramham, 2014).

BDNF is one of the most commonly studied proteins in memory research. In fact, in the last two decades plenty of studies described the participation of this neurotrophin in the acquisition, consolidation and long-lasting storage of different memory types (Ou et al., 2010; Martínez-Moreno et al., 2011; Bekinschtein et al., 2014). In particular, the role of BDNF in memory extinction, a process induced by repeated non-reinforced reactivation resulting in an inhibitory memory that opposes the original learned response, is well documented (Peters et al., 2010; Andero and Ressler, 2012; Xin et al., 2014). Maybe because of that, BDNF involvement in memory reconsolidation has been less investigated, although recent studies indicate that BDNF does play an essential function in this process, too.

## Memories are Adaptable: the Reconsolidation Process

Müller and Pilzecker (1900) postulated that learning does not generate permanent memories instantly but these are initially vulnerable to disruption and become stable only after a period of consolidation. Almost 50 years later, Hebb (1949) proposed that recently acquired information persists during a short period as reverberant activity in local circuits. This resonance would induce structural changes in synapses of that network, allowing permanent memory storage. The idea that structural modifications underlie long-term memory gave rise to the synaptic consolidation hypothesis, which postulates that newly learned information is stored in the brain through a consolidation process that depends on gene expression and *de novo* protein synthesis for developing new synaptic connections and/or remodeling existing ones to support lasting memory storage (McGaugh, 1966).

The consolidation hypothesis posits that memories are immutable representations of the events that originated them. However, literature on experimental amnesia from the late 1960s conflicted with this view, pointing that memories can be altered upon reactivation. In those years, Donald Lewis and coworkers found that well-established fear memories could be impaired by electroconvulsive shock treatment given after a brief re-exposition to the conditioned stimulus (CS) that originated the conditioned fear response. Importantly, the same treatment was unable to affect retention when the reminder was omitted. The fear response also persisted in animals re-exposed to the CS alone, excluding the possibility that memory extinction could account for these results (Misanin et al., 1968). Based on these findings, it was suggested that retrieval induces the transition of memory from an inactive to an active state, and that interfering with this process could lead to memory loss (Lewis and Bregman, 1973; Lewis, 1979). Schneider and Sherman (1968) found similar results for avoidance memories, and it was later reported that administration of strychnine after aversive memory reactivation facilitated retention in rats (Gordon and Spear, 1973). Nevertheless, some studies failed to replicate Lewis’ group findings (Dawson and McGaugh, 1969; Squire et al., 1976), and hence, the consolidation hypothesis continued dominating the field of learning and memory for decades. This conceptual framework excluded the possibility that established memories were actively reprocessed during retrieval. Indeed, it took almost 25 years until Przybyslawski and Sara (1997) successfully reinstated the idea that memories are susceptible to updating by ongoing experiences as a mainstream hypothesis showing that NMDA receptors blockade following consolidated spatial memory reactivation induces persistent amnesia. These findings led Przybyslawski and Sara (1997) to propose that some biochemical pathways activated during consolidation are also necessary to reconsolidate the active trace destabilized upon retrieval, a hypothesis confirmed by Nader et al. (2000) using auditory fear conditioning as a learning paradigm. Since then, memory reconsolidation has been observed in numerous animal species, using experimental paradigms evaluating distinct memory types, and employing a plethora of pharmacological treatments and behavioral challenges able to modulate it (Nader, 2015). However, memory retrieval does not always trigger reconsolidation but several boundary conditions constrain the occurrence of this process. For example, depending on the learning paradigm, memory labilization upon retrieval can be contingent on novelty detection (Morris et al., 2006; Rossato et al., 2007), mismatch perception (Pedreira et al., 2004) or reactivation of conflicting information (Radiske et al., 2017a). In addition, it has been reported that old as well as strong memories are usually more resistant to reconsolidation blockers than new and/or weak ones (Milekic and Alberini, 2002; Eisenberg and Dudai, 2004; Suzuki et al., 2004), suggesting that different reactivation protocols might be required to destabilize deep-rooted, robust memories. Extinction induction can also restrain memory reconsolidation (Pedreira and Maldonado, 2003; Suzuki et al., 2004) and although reconsolidation and extinction are dissociable and reciprocally exclusive processes (Merlo et al., 2014) they share several neurotransmitter systems and intracellular signaling pathways (Cahill and Milton, 2019) and also can influence each other. Indeed, phenomena involving extinction within the reconsolidation window, as well as reconsolidation of reactivated extinction memory, have been described (Monfils et al., 2009; Rossato et al., 2010).

It has been proposed that memory reconsolidation would mediate incorporation of new information into previously stored representations to support mental schema reorganization (Sara, 2000; Hupbach et al., 2007; Rossato et al., 2007) or would maintain memory relevance by preventing forgetting and supporting the lingering systems consolidation process that gradually stabilizes memories (Dudai and Eisenberg, 2004; Alberini, 2011). These two hypotheses are not mutually exclusive but the former requires an initial memory destabilization stage while the latter does not necessarily do so. Then, it could be expected that the molecular mechanisms responsible for restabilizing an updated memory differ from those involved in an ongoing consolidation process that evolves over time to strengthen the trace. However, our knowledge about the neurochemical bases of reconsolidation is still incipient. Difficulties do not rely only on identifying brain regions and intracellular pathways that might be differentially required for additional learning or memory modification through reconsolidation but on the fact that several neurotransmitter systems and signaling cascades that seem to be involved in reconsolidation are also required for other retrieval-induced cognitive processes, such as extinction (revised in Cahill and Milton, 2019). For example, extinction and reconsolidation are NMDA-dependent processes (Suzuki et al., 2004) modulated by dopaminergic and endocannabinoid neurotransmission (Marsicano et al., 2002; Hikind and Maroun, 2008; Lee and Flavell, 2014; Rossato et al., 2015) that involve AMPA receptor trafficking (Kim et al., 2007; Rao-Ruiz et al., 2011), all of which directly or indirectly control synaptic plasticity.

## BDNF and Memory Reconsolidation

Neurotrophins are key regulators of long-term synaptic modifications. They are synthetized and secreted in an activity-dependent manner, acting locally at active synapses to enhance neurotransmission efficacy (Canossa et al., 1997; Poo, 2001). In particular, BDNF synthesized at dendrites is critical for LTP, a form of long-term plasticity and a putative cellular mechanism for memory storage (Morris et al., 1986), mediating post-translational modifications at pre- and post-synaptic terminals and regulating local translation. BDNF contributes also to structural changes in synaptic spines (Tanaka et al., 2008) and sustains LTP even when protein synthesis is inhibited (Pang et al., 2004). Reactivation of potentiated synapses can render LTP sensitive to protein synthesis inhibition once again, indicating that LTP stability is a function of neuronal activity level (Fonseca et al., 2006) and suggesting that molecular mechanisms involved in LTP might also be important for reconsolidation. It is not surprising then that BDNF can also mediate reconsolidation-induced plasticity helping to remodel synapses activated by retrieval without affecting other circuits. In fact, memory reconsolidation depends on several molecules involved in LTP maintenance, such as Zif-268 and PKMζ (Lee et al., 2004; Rossato et al., 2019). In this respect, Samartgis et al. (2012) found that BDNF administration following a reminder session facilitates avoidance memory in chickens, showing for the first time that BDNF is indeed necessary for memory reconsolidation. In agreement with these results, reactivation-induced fear conditioning memory enhancement requires hippocampal BDNF expression in stressed rats (Giachero et al., 2013). Also, post-retrieval intra-CA1 spermidine administration lengthens contextual fear memory duration through a mechanism that depends on hippocampal BDNF maturation as well as on the interaction between this neurotrophin and its main receptor, tropomyosin-related receptor kinase B (TrkB; Signor et al., 2017). Further, increased BDNF mRNA and protein levels as well as TrkB activation in the insular cortex accompany hippocampus-independent conditioned taste aversion (CTA) memory retrieval, and interfering with BDNF synthesis in this cortex after reactivation causes amnesia. Notably, post-retrieval intra-insular cortex BDNF administration reverses CTA impairment and enhances weak CTA memory retention (Wang et al., 2012). Reactivation of fear extinction memory also increases BDNF levels and TrkB phosphorylation in the rat hippocampus while intra-CA1 administration of function-blocking anti-BDNF antibodies after extinction memory retrieval hampers extinction memory reconsolidation causing reinstatement of the extinguished fear. Importantly, hippocampus BDNF signaling activation preserves the learned extinction response when extinction memory reconsolidation is blocked (Radiske et al., 2015). This suggests that the mnemonic representation that controls behavior during retrieval is the one that gets weaken, as proposed by the trace dominance theory (Eisenberg et al., 2003), and also that BDNF signaling is sufficient to reconsolidate the prevailing memory. In line with these results, BDNF Val66met polymorphism, which is associated with hippocampus plasticity and BDNF trafficking (Egan et al., 2003), impairs conditioned fear memory storage when a brief fear reactivation session is followed by extended extinction training in humans (Asthana et al., 2016).

**FIGURE 1:**
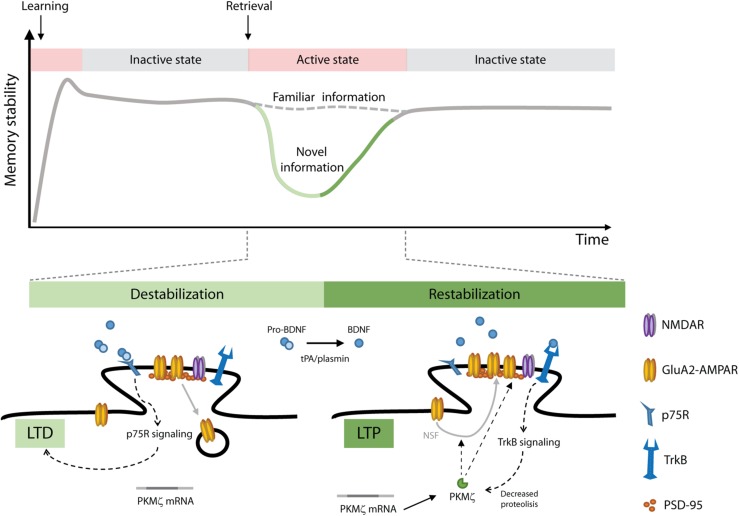
A model of object recognition memory (ORM) reconsolidation. After learning, memories are gradually stabilized through consolidation. Consolidated memories are activated upon retrieval and depending on the conditions prevailing during this process (i.e., perception of novel information) they can be destabilized allowing their modification. To persist, updated memories must be restabilized through reconsolidation. We propose that ORM destabilization involves pro-BDNF/p75R signaling and LTD induction in the hippocampus. Restabilization of the updated memory trace involves pro-BDNF proteolysis, BDNF/TrkB signaling, PKMζ upregulation and insertion of GluA2-containing AMPAR into hippocampal postsynaptic densities. NSF, *N*-ethylmaleimide sensitive fusion protein; PSD-95, post synaptic density 95 protein; tPA/plasmin, tissue plasminogen activator/plasmin system.

The participation of BNDF in memory reconsolidation is not restricted to distressing memories. Recognition memory, a major component of declarative memories, provides the ability to identify previously encountered events, objects and individuals. In rats, object recognition memory (ORM) maintenance requires *de novo* hippocampal protein synthesis after retrieval, but only when novelty is perceived during reactivation, suggesting that reconsolidation recruits the hippocampus to incorporate new information into the active recognition trace (Rossato et al., 2007). In neurons, BDNF is synthetized as a precursor peptide, proBDNF, which is stored or further cleaved to produce mature BDNF (Pang et al., 2004; Hwang et al., 2005). In the hippocampus, pro and mature forms of BDNF are abundant in presynaptic terminals of glutamatergic neurons and after release they act locally through their binding to p75 neurotrophin receptor (p75R) or TrkB, respectively. Activation of p75R by proBDNF facilitates LTD at CA1 synapses (Woo et al., 2005), and proBDNF extracellular conversion to BDNF by the tissue plasminogen activator (tPA)/plasmin system is essential for sustaining LTP (Pang et al., 2004). In accordance with these observations, ORM reconsolidation modifies hippocampal synaptic efficacy in rats, inducing a rapid depotentiation phase that occurs around 1.5 h after retrieval and is followed by a synaptic potentiation stage taking place ∼4.5 h thereafter (Clarke et al., 2010). Consistent with these findings, ORM reconsolidation is also accompanied by post-retrieval proteolysis of proBDNF, which augments BDNF levels and promotes functional BDNF/TrkB interaction in the hippocampus to restabilize the reactivated representation and incorporate new declarative information concurrently (Radiske et al., 2017b). PKMζ is a constitutively active PKC isoform highly expressed in the hippocampus. that would be responsible for sustaining long-term memory storage (Sacktor, 2008). BDNF modulates PKMζ turnover (Kelly et al., 2007) and maintains PKMζ-dependent late-LTP in the hippocampus even in the absence of protein synthesis (Mei et al., 2011). Interestingly, we recently demonstrated that BDNF mediates ORM reconsolidation-induced plasticity through PKMζ, which, in turn, regulates AMPAR trafficking at postsynaptic densities in the dorsal hippocampus to update the reactivated memory trace (Rossato et al., 2019). Figure 1 shows a model of the molecular mechanism that might be mediating ORM reconsolidation. The study of Rossato et al. (2019) also provides behavioral, pharmacological and electrophysiological evidence supporting the idea that disrupting the reconsolidation process causes memory erasure. Importantly, blocking BDNF maturation as well as inhibition of BDNF downstream effectors after retrieval delete the reactivated recognition memory trace but leave dormant ORM intact, suggesting that memory destabilization specifically affects reactivated synapses and that BDNF modulates local synaptic remodeling to restabilize the updated trace. Notwithstanding this, other studies found that BDNF involvement in memory processing is restricted to memory consolidation and plays no role in reconsolidation (Lee et al., 2004; Barnes and Thomas, 2008; Lee and Hynds, 2013). This discrepancy must be due to the fact that most of these studies employed pre-training or pre-reactivation infusions of BDNF antisense oligodeoxynucleotides to hinder BDNF expression by knocking down proBDNF mRNA translation but were unable to affect the conversion of already available proBDNF to mature BDNF, which is essential for memory reconsolidation (Radiske et al., 2015).

## Implications of Bdnf Signaling Manipulation During Memory Reconsolidation

Determining the molecular basis of retrieval-induced cognitive processes is necessary not only to understand the dynamics of the memory storage process but also to prevent memory decline and treat disorders associated with the persistent reenactment of maladaptive recollections (Kida, 2019). The studies reviewed here suggest that targeting BDNF is a promising adjuvant to help patients recontextualize disturbing memories during reconsolidation-based therapies.

Memories about emotionally arousing events are usually persistently stored which, in some cases, lead to intrusive and distressing recollections that may result in anxiety, phobia or other types of disarrays such as post-traumatic stress disorder (PTSD; Ehlers, 2010). A potential tool to treat the exacerbated avoidance responses caused by the expression of fear memories at the core of some phobic behaviors is to disrupt its reconsolidation (Beckers and Kindt, 2017). However, this intervention presents limitations because, as mentioned above, retrieval does not always induce memory destabilization. Extinction-based psychotherapies are an alternative strategy to reduce traumatic memory expression, but their effects are not persistent and the current challenge is to maintain the extinction memory over time (Vervliet et al., 2013). In this respect, it was recently proposed that enhancing reconsolidation of extinction memory could be a viable strategy to avoid its decay (Radiske et al., 2015; Rosas-Vidal et al., 2015). Findings from the last decade show that BDNF modulates reconsolidation of both aversive and extinction memories (Wang et al., 2012; Giachero et al., 2013; Radiske et al., 2015; Signor et al., 2017). Overall, these studies suggest that drugs interfering with BDNF signaling during reconsolidation of aversive memories could help to impair its retention, while approaches that activate BDNF/TrkB pathways after extinction memory retrieval may promote its persistence, preventing reappearance of the fear response.

Antidepressants can influence BDNF levels bidirectionally. For example, a single dose of the serotonin re-uptake inhibitor fluoxetine decreases BDNF expression (Coppell et al., 2003) and attenuates aversive memory persistence in rats (Slipczuk et al., 2013), but chronic administration of the same agent upregulates BDNF mRNA levels (Coppell et al., 2003). These observations suggest that the patient’s medication history can deeply influence the outcome of therapies based on memory reconsolidation. Acute interventions with antidepressants after traumatic memory reactivation may disrupt its reconsolidation, helping to reduce the disturbing symptoms. On the other hand, reconsolidation interference should be avoided in patients that are being treated for depression with drugs able to augment BDNF function. In this case, extinction-based treatments could be more effective, since enhanced BDNF signaling promotes fear memory extinction (Peters et al., 2010) as well as its persistence through memory reconsolidation (Radiske et al., 2017a).

An alternative strategy that is being study to enhance PTSD exposure therapy efficacy consist of coupling extinction sessions with physical exercise, which increases peripheral BDNF levels (Powers et al., 2015). In this respect, it has been recently shown that lactate mediates the facilitatory effect of physical exercise on cognition by upregulating hippocampal BDNF expression (El Hayek et al., 2019). Because the healthy human brain can uptake systemically administered lactate (van Hall et al., 2009), it would be interesting to evaluate lactate as a putative therapeutic molecule to reduce fear relapse by potentiating extinction through the enhancement of extinction memory reconsolidation.

Alzheimer’s disease (AD) progression has also been associated with impaired reconsolidation and reduced BDNF signaling (Hock et al., 2000; Ohno, 2009), suggesting that increasing BDNF function during reconsolidation could partially counteract declarative memory deficits in AD patients. In this respect, transcranial direct current stimulation, which activates signaling downstream BDNF and elicits LTP-like mechanisms in rats, improves episodic and semantic memories in AD patients (Cocco et al., 2018) a result in line with earlier findings showing that the blood-brain barrier permeable TrkB agonist 7,8-dihydroxyflavone (7,8-DHF) ameliorates cognitive decline in AD animal models (Devi and Ohno, 2012).

## Concluding Remarks

The studies reviewed in this article suggest that BDNF mediates enduring synaptic changes required for memory strengthening and updating upon retrieval. Several pharmacological and non-pharmacological approaches to modulate BDNF signaling and expression are currently under consideration, and exposure-based psychotherapy could take advantage of those findings. However, future research should also consider BDNF signaling interaction with other mediators of memory reconsolidation. An important issue that limits the effectiveness of reconsolidation-based treatments is that retrieval does not always induce memory destabilization. However, it was recently proposed that memory destabilization can be enhanced pharmacologically (Lee and Flavell, 2014), offering the opportunity to improve familiar declarative memories or treat old traumas that are usually resistant to reconsolidation.

## Author Contributions

MG and AR conceptualized the review, performed the literature research, interpreted the data, and wrote the manuscript. MC contributed to the data interpretation, edited parts of the manuscript, and critically revised and approved its final version.

## Conflict of Interest Statement

The authors declare that the research was conducted in the absence of any commercial or financial relationships that could be construed as a potential conflict of interest.
